# Endomembranes: Unsung Heroes of Mechanobiology?

**DOI:** 10.3389/fbioe.2020.597721

**Published:** 2020-10-22

**Authors:** Santosh Phuyal, Francesco Baschieri

**Affiliations:** ^1^Department of Molecular Medicine, Institute of Basic Medical Sciences, University of Oslo, Oslo, Norway; ^2^Inserm U1279, Gustave Roussy Institute, Université Paris-Saclay, Villejuif, France

**Keywords:** mechanobiology, mechanotransduction, endomembranes, endoplasmic reticulum, Golgi

## Abstract

Mechanical stimuli have profound effects on the cellular architecture and functions. Over the past two decades, considerable progress has been made in unraveling the molecular machineries that confer cells the ability to sense and transduce mechanical input into biochemical signals. This has resulted in the identification of several force-sensing proteins or mechanically activated ion channels distributed throughout most cell types, whereby the plasma membrane, cytoskeleton, and the nucleus have garnered much attention. Although organelles from the endomembrane system make up significant portion of cell volume and play pivotal roles in the spatiotemporal distribution of signaling molecules, they have received surprisingly little attention in mechanobiology. In this mini-review, we summarize results that document participation of the endomembrane system in sensing and responding to mechanical cues.

## Introduction

Cells in our body are continuously subjected to mechanical perturbations, to which they respond. From a physical perspective, one can draw parallels between the cell and a tensegrity structure, a term that refers to a system relying on pre-stressed (under tension) components balanced by compressed elements (Ingber et al., 2014). In the cellular context, tensegrity is supported mainly by the cytoskeleton, with actin filaments being the primary tense structures, balanced by the compression-bearing microtubules (Ingber et al., 2014). More recently, a tensegral role for the plasma membrane (PM) was also documented (Diz-Muñoz et al., 2013; Pontes et al., 2017). Tensegral structures quickly balance external physical forces by reversibly rearranging their shape. Accordingly, cells subjected to tensile, compressive, or shear stresses show cytoskeletal rearrangements (Discher et al., 2005) and fluctuations in the PM tension (Le Roux et al., 2019). Such changes in turn affect several behaviors of the cell, ranging from cell motility and endocytosis (Keren et al., 2008; Houk et al., 2012; Thottacherry et al., 2018; Baschieri et al., 2020a) to cell fate determination (McBeath et al., 2004; Przybyla et al., 2016; Belly et al., 2019).

**FIGURE 1:**
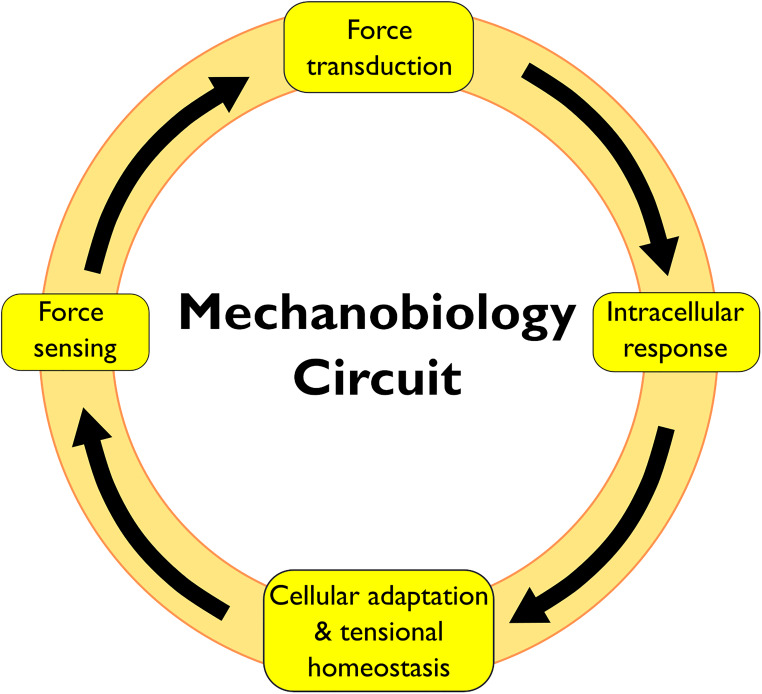
Schematic of a mechanobiology circuit.

The cellular response to physical forces proceeds in four phases: (i) force sensing, (ii) force transduction, (iii) intracellular response, and (iv) cellular adaptation and restoration of tensional homeostasis (Figure 1). The phases of this mechanobiology circuit involve molecules distributed throughout the cell, which demand a coordinated action of multiple cellular structures. However, mechanobiology research has focused largely on the cytoskeleton, the PM and the nucleus, but only scant attention was directed to other cellular structures (Discher et al., 2005; Janota et al., 2020). The endoplasmic reticulum, the Golgi, the endosomes and the autophagosomes are all directly linked to the cytoskeleton and could thus directly sense/transduce forces. Additionally, several mechanosensitive proteins are distributed along the membranes of these organelles.

Over the course of this review, we will highlight the current knowledge on the mechanobiology of these endomembrane compartments.

## Endoplasmic Reticulum

In eukaryotic cells, the endoplasmic reticulum (ER) is one continuous, complex membrane system characterized by interconnected tubules and sheets, which extend throughout the cytosol covering more than 20% of the cell volume (Bolender, 1974; Wiest et al., 1990; Voeltz et al., 2002). Even though the ER is part of the nuclear envelope, we will not focus here on the mechanobiology of the nucleus. We address interested readers to other reviews (Aureille et al., 2017; Maurer and Lammerding, 2019). The ER plays an instrumental role in protein synthesis, folding and quality control, lipid synthesis, and Ca^2+^ homeostasis. As the first station of the secretory pathway, it provides budding platforms for COPII coated vesicles that ensure delivery of proteins and lipids to other organelles in the cell (McCaughey and Stephens, 2019). The ER is also a target of signals emanating from extracellular and intracellular stimuli, and of autoregulatory signals from the organelle itself (Centonze and Farhan, 2019).

The highly dynamic nature of the ER-network reflects its adaptability and responsiveness to intracellular and extracellular changes. It undergoes constant rearrangements from tubule branching to fusion (Lee and Chen, 1988), to expansion (Wiest et al., 1990). Its widespread and dynamic morphology allows the ER to establish physical contacts with almost all the other cellular organelles (Wu et al., 2018) thereby making this endomembrane compartment a putative platform for force transmission inside the cell.

Despite these unique morphological features, only a few studies have investigated the relationship between mechanical cues and the ER. Nevertheless, recent evidence suggests that the ER harbors mechanosensitive and mechanotranducing elements (Nakayama et al., 2012; Kim et al., 2015; Lee et al., 2020; Nava et al., 2020).

**FIGURE 2:**
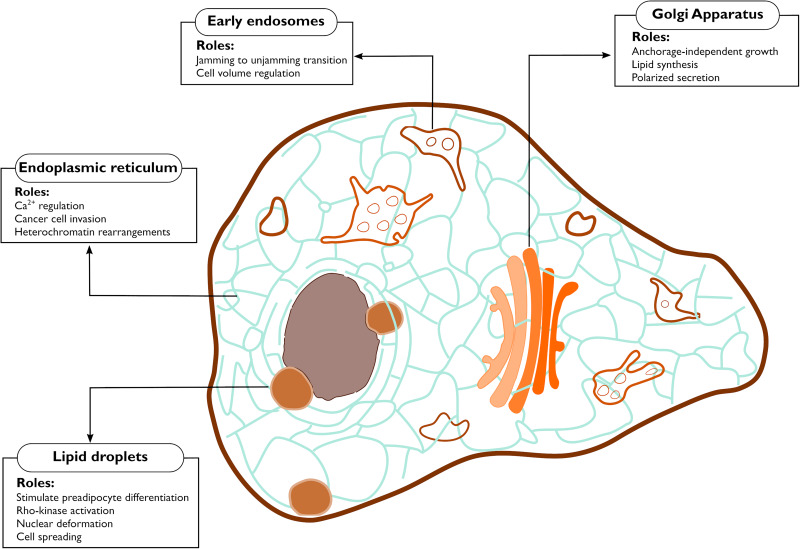
Functions controlled by mechanical properties of the endomembranes. The figure highlights the cellular processes debated in this review that are affected by mechanical changes occurring at endomembranes.

Mechanosensitive channels are present in the ER in fission yeast (Nakayama et al., 2012) and in mammalian cells (Lee et al., 2020). In fission yeast, hypo-osmotic shock-induced cell swelling activated Msy1 and Msy2, two proteins that localize to the perinuclear and the cortical ER, respectively. These proteins played critical roles for cell survival by regulating cell volume and intracellular Ca^2+^ levels (Nakayama et al., 2012). In mammalian cells, the mechanosensitive Pannexin 1 (PANX1) was previously shown to be localized in both the PM and the ER (Vanden Abeele et al., 2006). At the PM, PANX1 organizes into non-junctional hemichannels that are responsive to mechanical input and permeable to ATP (Bao et al., 2004; Dahl, 2018). Recently, Lee et al. (2020) demonstrated that PANX1 at the ER is also mechanosensory and functionally distinct from the PM localized PANX1. The ER-localized PANX1 channel responded to ultrasound stimulations in invasive cancer cells causing the release of Ca^2+^ from the ER (Figure 2). This response involved neither the cytoskeleton nor the PM PANX1 pool, thereby pointing toward a direct response by the ER pool of PANX1 (Lee et al., 2020). The Ca^2+^ channel Piezo1, localized both at the PM and at the ER (McHugh et al., 2010; Gudipaty et al., 2017), was also found to respond to cell stretching from both locations (Nava et al., 2020). Skin epidermis stem cells soften their nucleus in response to stretching in order to avoid DNA damage (Figure 2). Intriguingly, such response is dependent on ER-localized Piezo1, which is activated by an increase in ER membrane tension, hence releasing Ca^2+^ (Nava et al., 2020).

Hinting for a broader role of the ER in mechanotransduction, fragmentation of the ER network impaired meiotic cytoplasmic streaming, a phenomenon wherein collective movement of cytoplasm occurs even in the absence of preexisting polarity, in *C. elegans* zygotes (Kimura et al., 2017). The study provided conclusive evidence that the ER is instrumental in propagating the force within the cytoplasm and dictating the alignment of the microtubules to drive meiotic cytoplasmic streaming (Kimura et al., 2017). This raises the intriguing question that an intact ER is necessary to properly integrate a mechanical response, which will need to be addressed in the future.

In other cases, *de novo* organelles derived from the ER may alter mechanical properties or mechanosensing of cells. For example, lipid droplets (LDs) are fat storage organelles that originate from the ER (Walther et al., 2017). LDs are stiffer than cell cytoplasm, and their expansion during adipogenesis mechanically distorts the intracellular environment consequently stiffening the cell (Figure 2; Shoham et al., 2014). Studies have shown that constant mechanical strain arising from adipocytes hypertrophy acts as a stimulus for preadipocyte differentiation (Ben-Or Frank et al., 2015), Rho-kinase activation (Hara et al., 2011), as well as cell spreading and cytoskeleton rearrangements (Chin et al., 2020). Stiffness resulting from adipocyte expansion also puts constant pressure on the extracellular matrix, and possibly acts as a mechanical cue for extracellular matrix remodeling. These findings highlight the fact that mechanical stimuli can equally originate within the cells, triggering the mechanical circuit (Figure 1) in an inside-out fashion. Whether other processes happening at the ER, such as (mis-)folded protein aggregation also acts as inside-out mechanical cue remains a topic to be explored.

## Golgi Apparatus

Most secretory proteins leaving the ER reach the Golgi Apparatus, where they undergo post-translational modifications. The mammalian Golgi Apparatus is a compact, ribbon shaped organelle usually found in proximity to the nucleus whose primary functions concern protein modification, and lipids and proteins trafficking (Rodriguez-Boulan and Müsch, 2005). Structurally, it consists of flat cisternae piled on each other organized into cis-, medial-, trans- stack, and ultimately, the Trans-Golgi-Network (TGN), with each region hosting specific enzymes to modify the secreted proteins (Potelle et al., 2015). Cargos traffic between the ER and the Golgi in coatomer (COPI or COPII) coated vesicles. Once at the TGN, cargos are packed into AP1 coated vesicles and delivered to their final destination (for more details see Lee et al., 2004; De Matteis and Luini, 2008; Glick and Luini, 2011).

Actin and microtubules (MTs) are both instrumental for vesicle trafficking and for the maintenance of the Golgi architecture (Egea et al., 2013; Fourriere et al., 2020; Ravichandran et al., 2020). Hence, the Golgi and its processes are intimately linked to the cytoskeleton, and are sensitive to cytoskeletal perturbations, suggesting a potential role of this organelle in the mechanobiology circuit (Figure 1). A first indirect evidence for such conjecture was provided 20 years ago, when a hyperactive mutant of Cdc42 was shown to interact with COPI and increase ER to Golgi transport, resulting in cellular growth in the absence of adhesion (Figure 2; Wu et al., 2000).

Several lines of evidence couple adhesion and Golgi Apparatus. Cell spreading upon adhesion triggers exocytosis of Golgi-derived vesicles delivering lipids required to expand the cell area (Gauthier et al., 2011). Cells in suspension display a fragmented Golgi already few minutes after losing contact with their substrate, a phenomenon that can be rescued by restoring Arf1 activity, which normally occurs downstream of activated integrins (Singh et al., 2018). Integrins are the main adhesion receptors of cells, and this observation suggests that the Golgi responds to a mechanotransduced signal downstream of integrins (Singh et al., 2018). Intriguingly, Golgi fragmentation in suspended cells was correlated with an increase in glycosylated proteins at the PM which did not depend on newly synthesized proteins, but rather on increased trafficking from the Golgi or increased Golgi-glycosylation activity (Singh et al., 2018). Hence, signaling originated from cellular adhesion could also affect the glycosylation function of the Golgi, but experimental evidence will be needed to confirm this speculation. These results frame the Golgi amidst the actors of a mechanotransduction pathway starting with cell adhesion, although it is still not clear whether the role of the organelle in this process is a passive or an active one.

Further strengthening the connection between Golgi and cell adhesion, vesicle fission from the TGN as well as Golgi architecture were shown to be regulated by RhoA (Zilberman et al., 2011; Eisler et al., 2018), a small GTPase tightly linked to cell contractility (Ridley and Hall, 1992; Burridge and Wennerberg, 2004). MT depolymerization by nocodazole causes the release of GEF-H1, a RhoA activator, and activated RhoA in turn leads to fission of TGN vesicles (Eisler et al., 2018). While MTs depolymerization is an artificial situation, a physiological pathway involving the focal adhesion localized protein KANK, a MTs capturing protein, was described to trigger GEF-H1 release without the need to depolymerize MTs (Rafiq et al., 2019). Considering that post-Golgi vesicles traveling on MTs are secreted at hotspots near cellular adhesions (Figure 2; Fourriere et al., 2019), it would be of interest to determine whether Golgi and adhesion structures can influence each other and whether this putative connection plays any role in mechanosensing and mechanotransduction.

Intriguingly, evidence suggests that the Golgi is mechanosensitive. Stiffness of the Golgi decreased upon actin depolymerization. Conversely, high acto-myosin contractility increased the organelle’s rigidity (Guet et al., 2014). Furthermore, direct application of a force to the Golgi reduced the number of vesicles leaving the TGN, indicating that forces alter physical properties and functions of the Golgi (Guet et al., 2014). More recently, the Golgi was found to induce changes in the lipid metabolism in response to extracellular forces (Romani et al., 2019). The sterol regulatory element-binding proteins (SREBPs) are transcription factors responsible for the transcription of genes involved in lipid synthesis (Shimano and Sato, 2017). SREBPs are kept inactive at the ER and get activated by proteolytic cleavage upon translocation to the Golgi. Such translocation is blocked by the phosphatase Lipin-1 in an Arf1 dependent fashion and Romani et al. (2019) showed that reducing acto-myosin contractility inhibits Lipin-1 activity at the Golgi (Figure 2). Importantly, the stiffness of the Golgi was found to be coupled to the stiffness of the ECM, implying that a force transmitted to the Golgi is at the basis of this regulation of lipid metabolism.

## Endo/Lysosomal System

The endo/lysosomal system is constituted of pleomorphic membranous carriers that mediate exchange of material between the cell and its extracellular environment. Progressive invagination of the PM lipid bilayer generates an early endocytic vesicle, which gets released in the cytoplasm (Doherty and McMahon, 2009; Thottacherry et al., 2019). Virtually all known endocytic mechanisms have proved to be affected by physical parameters such as substrate stiffness (Baschieri et al., 2018), cell stretching (Sinha et al., 2011; Thottacherry et al., 2018), and compression (Ferguson et al., 2017; Baschieri et al., 2020a). Since our focus here is on endomembranes, we address interested readers to other reviews for a thorough discussion on the mechanobiology of the early phases of endocytosis at the PM (Nassoy and Lamaze, 2012; Lacy et al., 2018; Baschieri et al., 2020b).

All early endocytic vesicles undergo a series of maturation and transition to the late endosomes. The fission and fusion events that are a part of the maturation process, lead to cargo sorting into tubular extensions or intraluminar vesicles (ILVs) (Gautreau et al., 2014), processes mediated respectively by the ESCRT complex (Williams and Urbé, 2007) and Arp2/3 actin nucleators (Derivery et al., 2009). Early endosomes are generally highly mobile and more peripherally localized, while late endosomes/lysosomes are relatively immobile and cluster close to the Golgi (Neefjes et al., 2017). This spatial organization of endosomes is functional for their maturation and is instrumental for the fine tuning of receptor tyrosine kinases (RTK) which will be turned off by the phosphatases concentrated at the cell center (Neefjes et al., 2017). Endosome-originated signaling controls a plethora of cellular functions (Sadowski et al., 2009; Scita and Di Fiore, 2010; Phuyal and Farhan, 2019) and recently a putative mechanotransducing role for endosomes was unveiled in collectively migrating epithelia (Malinverno et al., 2017; Palamidessi et al., 2019). In epithelial tissues, groups of closely associated cells flow in a fluid-like fashion. The flow stops in a process called jamming, when the cells become too dense and compressed. Reactivation of the endosomal protein Rab5A was sufficient to restart the movement of the jammed epithelia (Figure 2; Malinverno et al., 2017). Such jamming-to-unjamming transition was due to an increased ERK signaling from endosomes and was shown to promote breast cancer cell invasion by increasing cell motility of densely packed cancer cells (Palamidessi et al., 2019). These observations demonstrate that endosomes are cell density sensors.

The spatial segregation of endosomes can also be influenced by adhesive cues (Schauer et al., 2010). Micropatterned cells show an asymmetry in transferrin and EGF endocytosis which is not due to an unequal distribution of the transferrin and EGF receptors at the PM, but rather to the actin cytoskeleton. In fact a mild actin disruption results in complete loss of such spatial segregation (Grossier et al., 2014). Intriguingly, this peculiar distribution of endosomes translates into asymmetrical signaling downstream of the EGF receptor (Grossier et al., 2014) which could be instrumental for the cells to interpret their surroundings (Schauer and Goud, 2014). Considering that endosomes respond to adhesive cues, it would be interesting to test whether they can also interpret changes in the mechanical properties of the substrates. In fact, a recent work showed that culturing bladder epithelial cells on soft surfaces resulted in increased endosomal escape of intracellular bacteria, implying that substrate stiffness could affect endosomal integrity via a yet unidentified mechanism (Moorthy et al., 2020).

There appears to be a strong connection between the endo/lysosomal system and membrane tension (King et al., 2020; López-Hernández et al., 2020; Mercier et al., 2020). An increase in the PM tension is balanced by the exocytosis of a subpopulation of recycling endosomes (Gauthier et al., 2009) while a burst of endocytosis occurs when the PM tension decreases (Shi and Baumgart, 2015; Loh et al., 2019). Additionally, endosomes play a fundamental role in the adaptation of the cell to osmotic stress, situation where the cell confronts itself with ionic imbalance, leading in turn to water influx or efflux and volume changes (Figure 2). Both hypo-osmolarity and hyper-osmolarity are well-known to affect membrane tension. Recycling endosomes translocate the ion channel NHE7 from the TGN to the PM in response to hyperosmotic stress. This translocation causes a disequilibrium in the cytoplasmic ions, the net result being an increase in the number of lysosomes (López-Hernández et al., 2020). Such increase in lysosomes number is instrumental to scavenge the protein aggregates that form as a consequence of hyper-osmolarity (Willermain et al., 2014; Hock et al., 2018).

## Autophagosomes

Several cellular stressors are known to induce autophagy, a catabolic pathway that has its roots in the secretory pathway (Farhan et al., 2017). Its function is to enclose damaged intracellular components in autophagosomes for their degradation by the lysosomal machinery. The various types and importance of the autophagic processes in maintaining proper cellular homeostasis have been thoroughly discussed elsewhere (He and Klionsky, 2009; Kawabata and Yoshimori, 2020; Melia et al., 2020).

While the link between autophagy and various chemical stressors has been well-documented, only a handful of studies have explored the possible impact of mechanical stress on autophagy.

King et al. (2011) noted an upregulation in the rate of autophagosomes formation in Dictyostelium cells exposed to compressive forces, a finding they corroborated in mammalian cells. Induction of autophagy was observed with application of continuous compressive forces (∼0.2 kPa) within physiological range in both Dictyostelium and mammalian cells, a response that gradually decreased and returned to basal level once cells adapted to the compression by remodeling their cytoskeleton (King et al., 2011). A similar finding was reported in human trabecular meshwork (TM) primary cells (Porter et al., 2014). An important function of the TM is to maintain intraocular pressure, which is sensitive to pressure gradients and fluid movement. Hence, the cells in TM must sense and rapidly adapt to changes in mechanical forces in order to avoid mechanical injury and maintain proper function. Porter et al. (2014) found quick induction of autophagy in these cells when exposed to biaxial static mechanical stretch, thereby potentially linking autophagy to cellular adaptation to stretch. Finally, BAG3, a member of chaperone-assisted autophagy (Arndt et al., 2010), is presumably capable of tension sensing, coordinating autophagosome formation, and transcription regulation during mechanotransduction, hence contributing to the protection of mechanically strained muscle tissue (Ulbricht et al., 2013). These studies suggest that cells deploy autophagy to cope with mechanical forces and preserve tissue structure and cellular activity.

## Concluding Remarks

In this review, we have highlighted the emerging role of the endomembrane system in force sensing and transduction. Although the concept of physical forces eliciting a biological response is more than a century old (Wolff, 1892), the field of mechanobiology has garnered attention only in the past two decades, owing to the development of enabling technologies (Roca-Cusachs et al., 2017; Goujon et al., 2019). Seminal works describing the impact of mechanical forces on cell differentiation (Engler et al., 2006; Dupont et al., 2011), transformation (Paszek et al., 2005) and on several other processes have highlighted the important interplay between mechanical and biochemical signals. Much of the current mechanobiology research investigates mechanosensing and mechanotransducing pathways at the PM and the cell cytoskeleton underneath (Bershadsky et al., 2006; Lim et al., 2010). However, mechanical forces are not restricted to the PM. On the contrary, they can penetrate much deeper into the cells, thereby affecting all intracellular organelles (Kim et al., 2015; Feng et al., 2019). With the exception of the nucleus (Aureille et al., 2017; Maurer and Lammerding, 2019), the mechanical properties of intracellular organelles and their participation in the propagation of mechanical signals remain largely unknown. Tools and sensors to probe and quantify forces within the cell have recently experienced tremendous advancements, and are likely to aid in the integration and the expansion of mechanobiology to endomembranes (Mohammed et al., 2019; Straková et al., 2020). As shown by the researches highlighted in this short review, the organelles that constitute the secretory pathway are more than a passive cargo delivery system. Their intricate link to the cytoskeleton, the PM and the nucleus, and their propensity to assemble into dynamic signaling platforms suggests that they could play a paramount role in coordinating cellular responses to mechanical perturbations (Figure 2). This warrants careful characterization of the mechanical properties of this pathway to gain further insight into cellular mechanobiology.

## Author Contributions

SP and FB conceived and prepared the manuscript. Both authors contributed to the article and approved the submitted version.

## Conflict of Interest

The authors declare that the research was conducted in the absence of any commercial or financial relationships that could be construed as a potential conflict of interest.
